# RANKL Is a Downstream Mediator for Insulin-Induced Osteoblastic Differentiation of Vascular Smooth Muscle Cells

**DOI:** 10.1371/journal.pone.0029037

**Published:** 2011-12-15

**Authors:** Ling-Qing Yuan, Jia-Hua Zhu, Hua-Wen Wang, Qiu-Hua Liang, Hui Xie, Xian-Ping Wu, Hua Zhou, Rong-Rong Cui, Zhi-Feng Sheng, Hou-De Zhou, Xiao Zhu, Guan-Ying Liu, You-Shuo Liu, Er-Yuan Liao

**Affiliations:** 1 Institute of Metabolism and Endocrinology, Second Xiang-Ya Hospital, Central South University, Changsha, Hunan, People's Republic of China; 2 Geriatric Department, Second Xiang-Ya Hospital, Central South University, Changsha, Hunan, People's Republic of China; 3 Institute of Aging and Geratology, Second Xiang-Ya Hospital, Central South University, Changsha, Hunan, People's Republic of China; Florida International University, United States of America

## Abstract

Several reports have shown that circulating insulin level is positively correlated with arterial calcification; however, the relationship between insulin and arterial calcification remains controversial and the mechanism involved is still unclear. We used calcifying vascular smooth muscle cells (CVSMCs), a specific subpopulation of vascular smooth muscle cells that could spontaneously express osteoblastic phenotype genes and form calcification nodules, to investigate the effect of insulin on osteoblastic differentiation of CVSMCs and the cell signals involved. Our experiments demonstrated that insulin could promote alkaline phosphatase (ALP) activity, osteocalcin expression and the formation of mineralized nodules in CVSMCs. Suppression of receptor activator of nuclear factor κB ligand (RANKL) with small interfering RNA (siRNA) abolished the insulin-induced ALP activity. Insulin induced the activation of extracellular signal-regulated kinase (ERK)1/2, mitogen-activated protein kinase (MAPK) and RAC-alpha serine/threonine-protein kinase (Akt). Furthermore, pretreatment of human osteoblasts with the ERK1/2 inhibitor PD98059, but not the phosphoinositide 3-kinase (PI3K) inhibitor, LY294002, or the Akt inhibitor, 1L-6-hydroxymethyl-chiro-inositol 2-(R)-2-*O*-methyl-3-*O*-octadecylcarbonate (HIMO), abolished the insulin-induced RANKL secretion and blocked the promoting effect of insulin on ALP activities of CVSMCs. Recombinant RANKL protein recovered the ALP activities decreased by RANKL siRNA in insulin-stimulated CVSMCs. These data demonstrated that insulin could promote osteoblastic differentiation of CVSMCs by increased RANKL expression through ERK1/2 activation, but not PI3K/Akt activation.

## Introduction

Arterial calcification is a significant predictor for cardiovascular events in patient with diabetes mellitus and end-stage renal disease. In these cases, the main cell type involved is the vascular smooth muscle cell (VSMCs) [1], [2]; however, the mechanisms for arterial calcification still remain unclear.

There is an increasing amount of evidence to suggest that medial arterial calcification is an active and tightly regulated process that resembles mineralization in bone [1], [2]. Key functional aspects of bone formation and remodeling are recapitulated in vascular calcification, and many bone-related factors such as osteoprotegerin (OPG), receptor activator of NF-κB ligand (RANKL), alkaline phosphatase (ALP), osteocalcin (OC) and runt-related transcription factor 2 (RUNX2) have been found in calcified areas of the arterial wall and VSMCs [3]–[5]. The discovery that OPG-deficient mice develop severe osteoporosis and arterial calcification [6], [7] and that RANKL directly increased VSMC calcification [8] provided the first clue that the RANKL/OPG/RANK axis could be an important autocrine/paracrine axis for vascular calcification.

Hyperinsulinemia is a clinic characteristic of type 2 diabetes as a result of insulin resistance. Several studies have demonstrated that insulin level and insulin resistance are positively correlated with arterial calcification [9]–[14]. Moreover, an animal study of Zucker diabetic rats, a hyperinsulinism model, increased arterial stiffness that was related to arterial calcification [15], [16]. However, the relationship between insulin and VSMCs calcification remains controversial [17], [18]. In the present study, we used calcifying vascular smooth muscle cells (CVSMCs), referred to as a specific subpopulation of VSMCs that could express osteoblastic phenotype gene and form calcification nodule spontaneously [19], [20], to elucidate the effect of insulin on osteoblastic differentiation of CVSMCs and the mechanisms involved.

## Results

### Effects of insulin on the expression of RANKL mRNA and protein in CVSMCs

**Figure 1:**
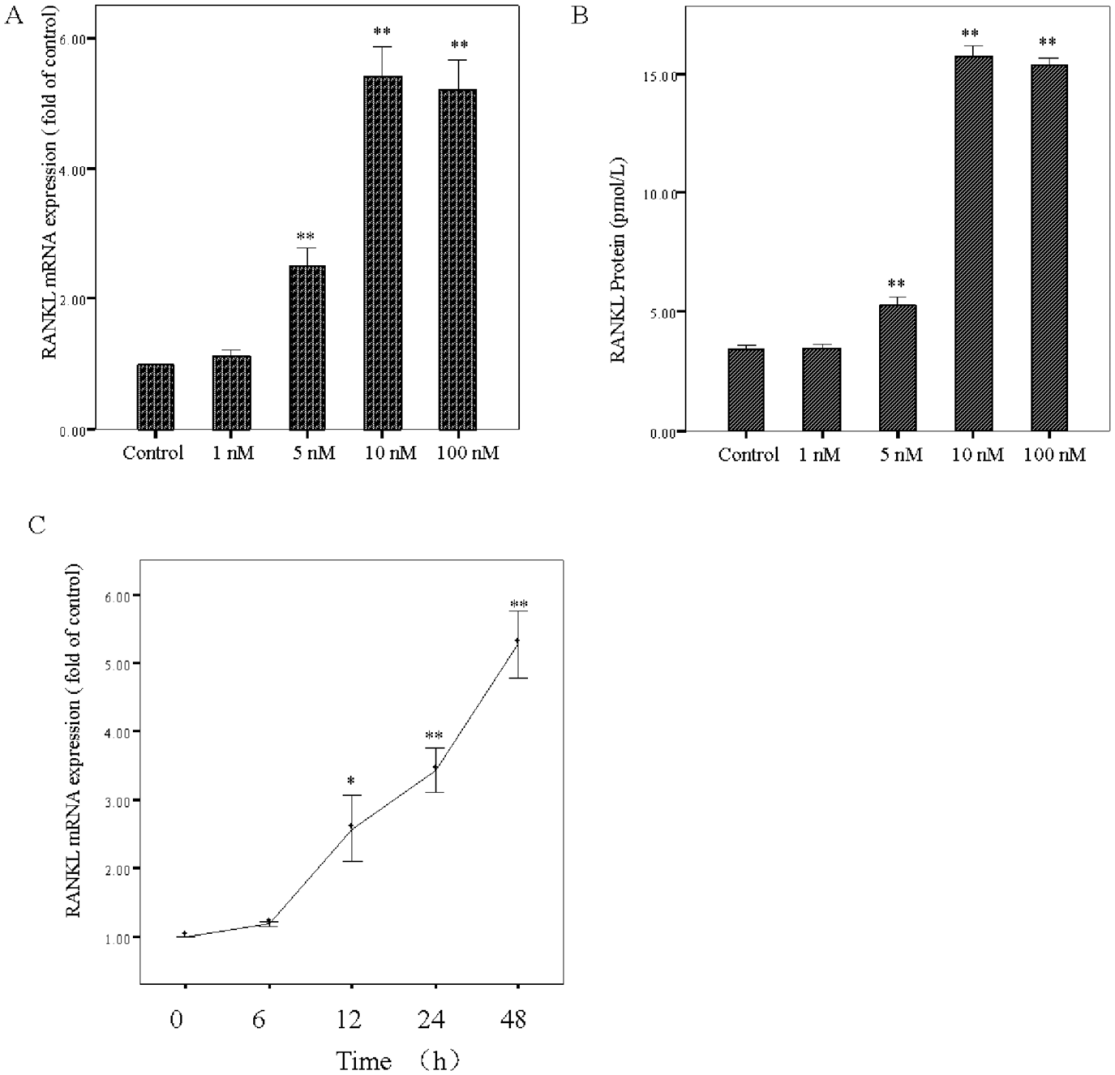
Effect of *in vitro* incubation of calcifying vascular smooth muscle cells (CVSMCs) with insulin on RANKL expression. Cells were exposed to vehicle or 1–100 nM insulin for 48 h or to 10 nM insulin for 6–48 h. (A) The dose–response of insulin on RANKL mRNA levels measured by real-time polymerase chain reaction (PCR) in cultured CVSMCs. Data are shown as percentage over basal values. (B) The dose–response of insulin on RANKL protein secreted by CVSMCs in the culture medium. (C) Time course of effects of 10 nM insulin on RANKL mRNA levels in cultured CVSMCs. (D) Time course of effects of 10 nM insulin on RANKL protein secretion in the culture medium by CVSMCs. The dots represent the percent of control RANKL levels at various time points. (*n* = 3, *p<0.05 vs. control, **p<0.01 vs. control.).

Physiologically, insulin concentrations *in vivo* may rise to ∼1 nM [21]. In the presence of insulin resistance, serum insulin concentrations may exceed these levels. We performed insulin dose–response and time course experiments to determine the effect of physiological and supraphysiological concentrations of insulin on mRNA levels and on RANKL protein secretion in CVSMCs. After 48 h, insulin at a 1 nM concentration had no effect on the expression of RANKL mRNA. RANKL mRNA levels increased significantly at 5 nM insulin stimulation, and the maximal effect of insulin was reached at 10 nM on RANKL mRNA levels. At markedly supraphysiological insulin concentrations there was a small decrease in RANKL mRNA levels compared with the maximum effect of insulin (Fig. 1A). RANKL protein secretion followed the mRNA trend (Fig. 1B). After 48 h of culture and at a concentration of 10 nM of insulin, expression of RANKL mRNA was greater than that of the controls (*p*<0.01) (Fig. 1C).

### Insulin activated ERK1/2 and Akt signaling pathway in CVSMCs

**Figure 2:**
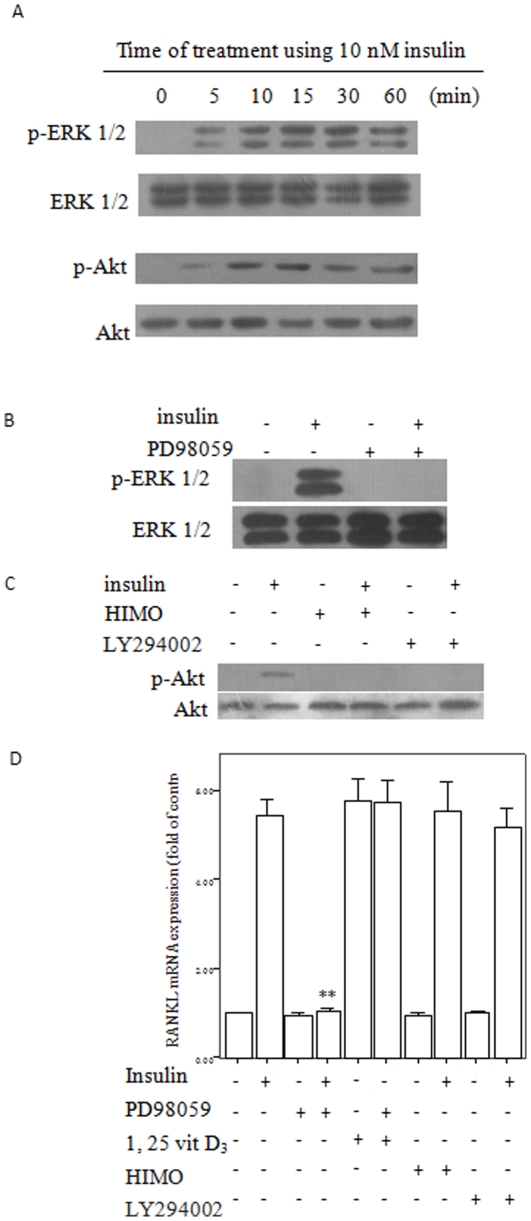
Insulin increased RANKL mRNA expression through ERK1/2, but not Akt signal pathway in calcifying vascular smooth muscle cells (CVSMCs). Cell lysates were subjected to western blot and incubated with anti-p-ERK1/2, ERK1/2, p-Akt, and Akt antibodies. (A) Effects of insulin on ERK1/2 and Akt activation in CVSMCs. Representative results are shown for cells exposed to 10 nM insulin for 5–60 min. (B) Cells incubated with PD98059 (10 µM) for 2 h prior to treatment with 10 nM insulin for 30 min. (C) Cells incubated with LY294002 (10 µM) or HIMO (10 µM) for 2 h prior to treatment with 10 nM insulin for 15 min. (D) Cells were incubated with PD98059 (10 µM), LY294002 (10 µM) or HIMO (10 µM) for 2 h before treatment with 10 nM insulin for 48 h. Cells were treated with 10^−7^ M 1α,25-dihydroxyvitamin D3 (1,25 vitD), or 1,25 vitD+10 µM PD98059 as another control. RANKL mRNA expression was determined by real-time quantitative PCR. Results are expressed as percent of control. Bars represent mean ± standard deviation (SD) (*n* = 3; **p<0.01 vs. insulin-treated control).

Recent studies have that demonstrated that ERK1/2 and Akt signaling pathways mediate insulin action [22], [23]. We examined if insulin induced ERK1/2 and Akt signaling in CVSMCs. As shown in Figure 2A, insulin stimulated the activity of a specific ERK1/2 in the CVSMCs after 5 min of incubation, when determined by the increase in the phosphorylated ERK1/2 levels the peak activation of ERK1/2 occurred at 15–30 min. Then, we examined whether insulin induced Akt signaling in CVSMCs. Insulin treatment increased phosphorylated Akt (p-Akt) levels after 5 min of incubation; the peak activation of Akt occurred at 15 min.

The activation of ERK1/2 and Akt by insulin was abolished by PD98059 (an inhibitor of ERK1/2), LY294002 (an inhibitor of PI3-K) or HIMO (an inhibitor of Akt), respectively (Fig. 2B and 2C). These data indicated that insulin stimulated ERK and PI3-K/Akt activation in CVSMCs.

### ERK1/2 signaling pathway mediated insulin-regulated RANKL expression in CVSMCs

Figure 2D showed that pretreatment of cells with the ERK1/2 inhibitor PD98059, but not the PI3K inhibitor LY294002 or the Akt inhibitor HIMO blocked the increasing RANKL mRNA expression stimulated by 10 nM insulin. To exclude any nonspecific effect of PD98059, LY294002 or HIMO, we used 1α,25-dihydroxyvitamin D3 (1,25 vitD) and 1,25 vitD+PD98059 as the control (both at 10^−7^ M) to induce RANKL mRNA expression. PD98059 did not block the increase in RANKL mRNA expression induced by 1,25 vitD.

### Insulin increased osteoblastic differentiation of CVSMCs and calcification of CVSMCs

#### Mineralization of matrix

**Figure 3:**
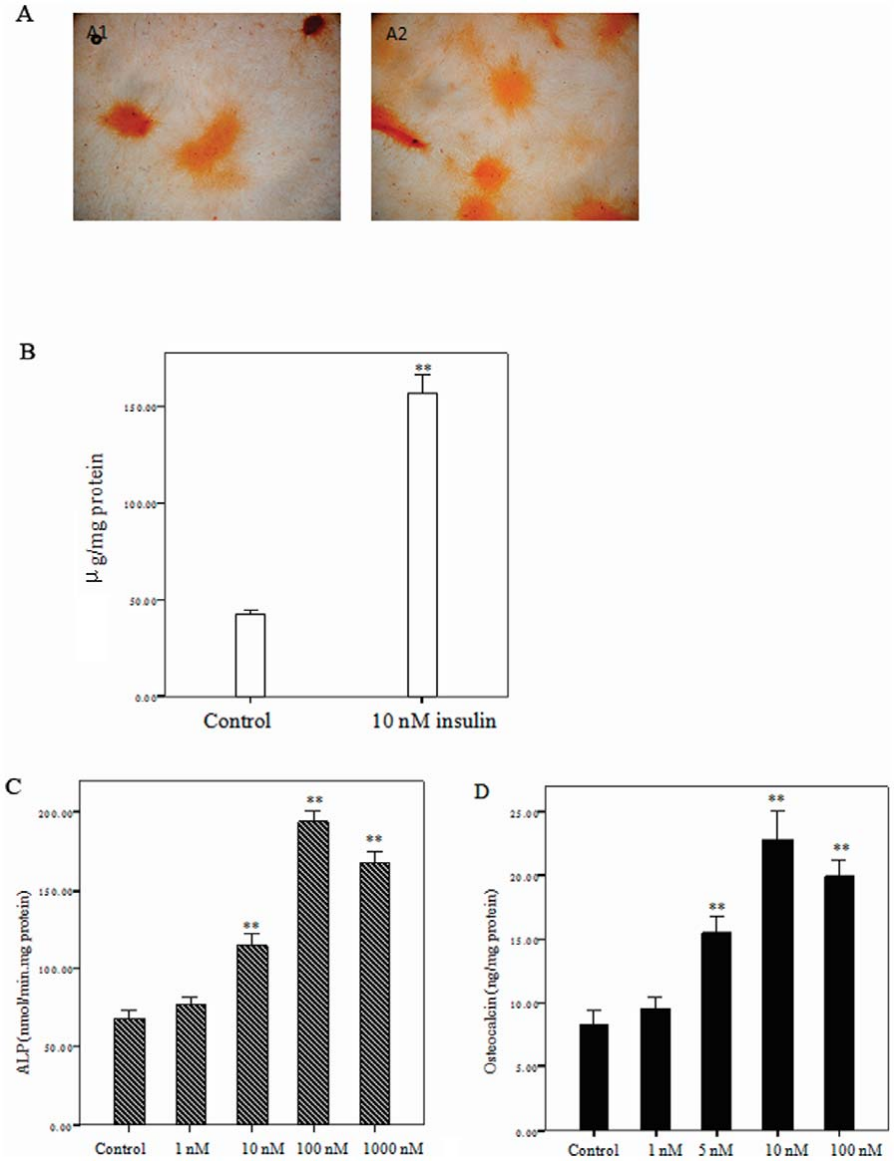
Insulin promoted the mineralization of the matrix in calcifying vascular smooth muscle cells (CVSMCs). CVSMCs were cultured in DMEM that contained 10% fetal bovine serum (FBS) in 24-well plates, in the presence of vehicle or 10 nM insulin for 20 days. Mineralization of the matrix was determined by Alizarin red S staining. (A) Representative microscopic view at a magnification of ×100. Left panel showed CVSMCs incubated with vehicle, and the right panel showed CVSMCs incubated with 10 nM insulin for 12 days. (B) Quantification of Alizarin red S stain through extraction with cetyl-pyridinium chloride. The amount of released dye was quantified by spectrophotometry at 540 nm. The bars represent mean ± standard deviation (SD) (*n* = 4; **p<0.01 vs. control). (C) Cells were treated with vehicle (serum-free medium) or insulin at 1, 5, 10, or 100 nM for 48 h in serum-free DMEM. ALP activity were determined and normalized to cell total protein. The dose–response of insulin on ALP activity in cultured CVSMCs. (D) Cells were treated with vehicle (serum-free medium) or insulin at 1, 5, 10, or 100 nM for 48 h in serum-free DMEM. Osteocalcin secretion were determined and normalized to cell total protein. The dose–response of insulin on osteocalcin secretion in cultured CVSMCs. The bars represent mean ± standard deviation (SD) (**p<0.01 vs. control, *p<0.05 vs. control, *n* = 4).

The calcification induced by insulin was also visualized by Alizarin Red S staining, as we show in Figure 3A. In this figure we show that incubation of CVSMCs with insulin increased the red staining that marks calcified areas. Figure 3B showed that insulin increased CVSMCs calcification as measured by CVSMC calcium levels.

#### ALP activity, osteocalcin secretion assay

Figure 3C showed the dose–response of effects of insulin on ALP activity in cultured CVSMCs. At insulin concentrations of 5, 10, and 100 nM, ALP activity increased markedly. Figure 3D shows the effects of insulin on osteocalcin secretion in cultured CVSMCs. Treatment with 5–100 nM insulin caused a significant increase in osteocalcin production and the maximal effect of insulin was reached after addition of 10 nM insulin.

### Involvement of RANKL and ERK1/2 signaling in insulin regulation of osteoblastic differentiation of CVSMCs

In order to illuminate whether and how RANKL, ERK1/2, and PI3K/Akt signaling was involved in the regulation of osteoblastic differentiation of CVSMCs, we used siRNA to block RANKL, and signal inhibitor to block ERK1/2, and PI3K/Akt signal pathways, respectively. Then, we observed ALP activity, which is an important phenotypic marker of osteoblastic differentiation.

**Figure 4:**
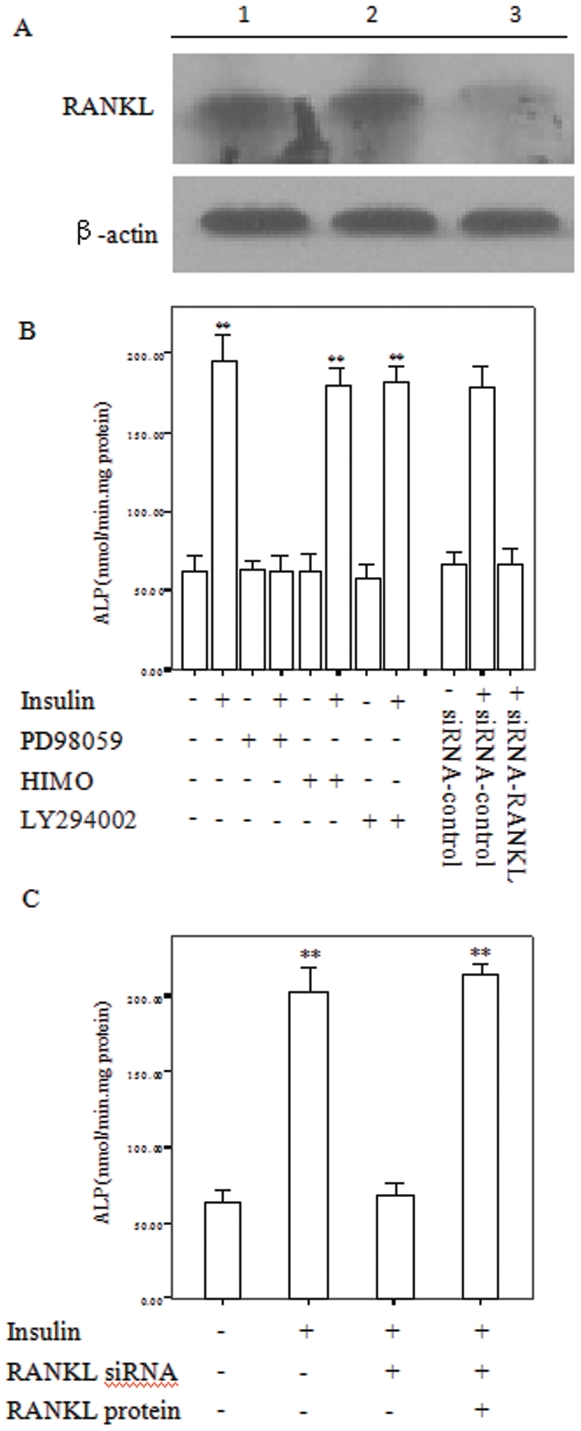
Insulin-promoted osteoblastic differentiation of calcifying vascular smooth muscle cells (CVSMCs) through the ERK1/2/RANKL signaling pathway. (A) Treatment with siRNA-RANKL blocked the expression of RANKL protein in osteoblasts. Cells were treated with siRNA control, or siRNA-RANKL. Total cellular protein was subjected to immunoblot analysis using anti-RANKL antibody. Lane 1, lysate from CVSMCs; lane 2, lysate from CVSMCs treated with siRNA control; lane 3, lysate from CVSMCs treated with siRNA-RANKL. (B) The cells were homogenized for ALP activity assay. Cells were incubated with PD98059 (10 µM), HIMO (10 µM) or LY294002 (10 µM) for 2 h before being treated with 10 nM insulin for 48 h. Cells were also treated with siRNA control or siRNA-RANKL in the presence of 10 nM insulin. (C) Effect of recombinant RANKL protein (15 pM) on the ALP activities decreased by RANKL-siRNA in 10 nM insulin-stimulated CVSMCs after 48 h. (*n* = 5; **p<0.01 vs. insulin-treated control).

Figure 4A showed that pretreatment of cells with the ERK1/2 inhibitor PD98059, but not the PI3K inhibitor LY294002 or Akt inhibitor HIMO blocked the increased ALP activity induced by 10 nM insulin. RANKL was knocked down efficiently by RNA interference in CVSMCs (Fig. 4A). Suppression of RANKL with siRNA-RANKL, but not control siRNA, also abolished the increased ALP activity indued by 10 nM insulin (Fig. 4B). Furthermore, 15 pM recombinant RANKL protein could recover ALP activities in the insulin-induced RANKL-knocked down CVSMCs (Fig. 4C). These data indicated that insulin-induced osteoblastic differentiation of CVSMCs is mediated by the ERK1/2/RANKL pathway.

## Discussion

VSMCs can express RANKL and recent results have shown that RANKL plays an important role in mediating arterial calcification [8], [24]. Our present data showed that insulin induced RANKL expression in CVSMCs through the ERK1/2 signal pathway, and these responses contributed to insulin-induced CVSMCs calcification. These findings suggested that RANKL induced by insulin in CVSMCs accelerated arterial calcification through an antocrine or a paracrine effect.

Extensive epidemiological data have shown that diabetes is correlated with arterial calcification and higher insulin level could predicate arterial calcification independently [11], [25]. The mechanisms of insulin involved in arterial calcification in these clinical settings are still controversial. Olesen *et al.'*s result demonstrated that insulin enhanced *in vitro*-induced calcification in VSMCs [17]. However, Wang *et al.* suggested that insulin attenuated VSMCs calcification in high phosphate medium [18]. These contradictory results might be explained by different cell types or different experimental condition. Increasing ALP activities, osteocalcin secretion is a marker of osteoblastic differentiation, and matrix mineralization is a hallmark of osteoblast phenotype. In our present study, we demonstrated that insulin upregulated ALP activities, increased osteocalcin secretion and promoted mineralization nodule formation. These results show that insulin stimulated osteoblastic differentiation of CVSMCs and increased CVSMC calcification, findings that are consistent with Olesen *et al.*'s results [17].

RANKL is a member of the tumor necrosis factor superfamily and is expressed mainly on osteoblasts and its immature precursors. RANKL binds to RANK and is expressed on the cell surface of hematopoietic osteoclast precursor cells, inducing osteoclastogenesis and activation and prolonging osteoclast survival by suppressing apoptosis. OPG acts as a decoy receptor by binding to RANKL and blocking its interaction with RANK, thus inhibiting osteoclast development. It has been suggested that the RANKL/OPG/RANK system is involved in the pathogenesis of arterial calcification. The first evidence for an involvement of OPG in arterial calcification was derived from OPG knockout mice that displayed severe osteoporosis and arterial medial calcification of the aorta and renal arteries. Furthermore, administration of OPG can inhibit vascular calcification in experimental animals [26], [27]. Olesen *et al.* showed that insulin could accelerate VSMC calcification and was accompanied by an increase in OPG expression [17]. However, their results did not explain if the altered OPG levels during the calcification could directly regulate the calcification process or if it was just a consequence of mineralization. Several studies have suggested that RANKL is involved in VSMC calcification. In addition, RANKL and RANKL transcripts could be detected in calcified arterial lesions from experimental animals [7], and RANKL expression was colocalized and upregulated in areas that displayed arterial calcification [28], [29]. In contrast, RANKL increased VSMC calcification by binding to RANK and increased BMP4 production through activation of the alternative NF-κB pathway [8]. Our recent results demonstrated that RANKL plays a pivotal role in omentin-attenuated arterial calcification in osteoprotegerin-deficient mice [24]. Our present data showed that insulin could increase the expression of RANKL and promote ALP activities in CVSMCs, concurrently. Furthermore, inhibition of RANKL expression by addition of siRNA abrogated the induction by insulin of CVSMC ALP activity. Then, recombinant RANKL protein recovered the ALP activity that was reduced by RANKL-siRNA addition in insulin-stimulated CVSMCs. These data suggest RANKL is the key regulator in insulin induced osteoclastic differentiation of VSMCs. In contrast, Tseng *et al.*'s result showed that upregulated RANKL induced by PKA in aortic smooth muscle cells could not mediate matrix calcification [30]. We think that these differences may result from use of a different cell model, as our previous study confirmed adenovirus-mediated over-expression of omentin-1 attenuated arterial calcification by decreased in RANKL expression in OPG knockout mice [24]. Thus we have shown that insulin regulated osteoblastic differentiation of CVSMCs through upregulation of RANKL in CVSMCs.

To gain further insight into the mechanisms by which insulin promoted RANKL expression in CVSMCs, we evaluated the signaling events. Recent studies have demonstrated the existence of two major signaling pathways, namely Akt and ERK1/2, which mediate insulin action [22], [23]. Signal transduction along both pathways is initiated by insulin binding to the insulin receptor. Transduction of insulin signal via the insulin receptor substrate (IRS) proteins PI3K and Akt is responsible for most, if not all, of the metabolic aspects of insulin action. The second signaling pathway, which involves Ras, Raf, and ERK1/2, is responsible for the mitogenic aspects of insulin action and is not involved in the mechanisms of metabolic insulin action [31], [32]. There are several independent signaling pathways documented that regulate RANKL expression in osteoblasts/stromal cells. These pathways include the ERK1/2 pathway (annexin II), the vitamin D receptor pathway [1], [25 vitD], the protein kinase A pathway (parathyroid hormone (PTH) and prostaglandin (PGE)2), the gp130 pathway (interleukin (IL)-6 and IL-11), and the calcium/protein kinase C pathway (high extracellular Ca^2+^) [33]–[37]. Here, we reported that insulin induced activation of Akt and ERK1/2 in CVSMCs. Furthermore, we demonstrated that pretreatment of cells with ERK1/2 inhibitor, but not PI3K inhibitor or Akt inhibitor, abolished the insulin-promoted RANKL expression in CVSMCs. These data indicated that insulin-induced RANKL expression was mediated by the ERK1/2 pathway.

In conclusion, the present data has provided evidence that insulin promoted osteoblastic differentiation of CVSMCs, and this effect is mediated by the ERK1/2/RANKL pathway. These findings suggest that insulin directly facilitates arterial calcification.

## Materials and Methods

### Reagents

Anti-rat extracellular signal-regulated kinase1/2 (ERK1/2), p-ERK, Akt, p-Akt antibody, anti a-actin polyclonal antibody, anti-RANKL, and rabbit IgG peroxidase conjugate antibodies were purchased from Santa Cruz Biotechnology (Waltham, MA, USA). The ERK 1/2 inhibitor PD98059, PI3K inhibitor LY294002 and Akt inhibitor 1L-6-hydroxymethyl-chiro-inositol 2-(R)-2-O-methyl-3-O-octadecylcarbonate (HIMO) was purchased from Calbiochem (San Diego, CA, USA). Recombinant rat RANKL was purchased from ABCAM (Cambridge, MA, USA).

### Cell culture

Six-week-old male Sprague Dawley rats were sacrificed, the aorta removed, and calcifying vascular smooth muscle cells (CVSMCs) were isolated as described previously [20], [38]. Briefly, the aortas were removed under sterile conditions. The aortas were rinsed several times in Hanks balanced salt solution, then the adventitia was removed and the aortas were minced and digested in 5 ml of digestion solution (0.125 mg/ml elastase, 0.25 mg/ml soybean trypsin inhibitor, 10 mg/ml collagenase I, 2.0 mg/ml crystallized bovine albumin, and 15 mM HEPES) at 37°C for 45 min. The cellular digests were filtered through sterile100-mM nylon mesh, centrifuged at 1000 rpm for 10 min, and washed twice in Dulbecco's modified Eagle's medium (DMEM) that contained 10% fetal bovine serum (FBS; Gibco-BRL Corp., NY, USA) before culture in the same medium. CVSMCs were isolated from cultures in which multicellular nodules appeared spontaneously. Cells were cloned by limiting dilution and single cell harvesting from these nodule-forming cultures. Cloned lines were identified as CVSMCs by their positive staining with monoclonal antibody α-actin, and by their ability to express high levels of ALP and to form calcified nodules. This investigation conforms to the Guide for the Care and Use of Laboratory Animals published by the US National Institutes of Health (NIH Publication No. 85-23, revised 1996). The approval was granted by the Second Xiangya Hospital of Central South University Ethics Review Board, China (No. 2010-S163).

### ALP activity, osteocalcin secretion assay in cultured CVSMCs

The cells were washed with PBS, and then the cells layers were scraped into solution. The lysates were homogenized. Then, ALP activity was assayed by spectrophotometric measurement of p-nitrophenol release at 37°C. ALP activity was normalized to total protein content of the cell layer. Osteocalcin released into the culture media was measured using a specific radioimmunoassay kit (DiaSorin Corp., Stillwater, MN, USA). To normalize protein expression to total cellular protein, a fraction of the lysate solution was used in a Bradford protein assay.

### Real-time quantitative PCR assay for RANKL mRNA expression

CVSMCs were plated in 25-cm^2^ flasks in DMEM containing 10% FBS. After 4 days of culture, cells were subsequently treated with vehicle (serum-free DMEM) or insulin at 1, 5, 10, or 100 nM for 48 h in serum-free DMEM. Cultures were also exposed to fresh serum-free medium with or without 10 nM insulin for 6–48 h.

SYBR green real time PCR assay was performed by Light-Cycler technology (Roche) in 20 µl PCR mixture volume consisting of 10 µl of 2× Quantitect SYBR Green PCR Master Mix (Qiagen), containing HotStarTaq DNA polymerase, 500 nMof each oligonucleotide primer and 5 µl of retrotranscribed cDNA. Preliminary experiments were carried out for primer concentration optimization. Primer sequences for RANKL were 5′-CATCGGGTTCCCATAAAG -3′ and 5′- GAAGCAAATGTTGGCGTA -3′. For GAPDH, the PCR primers were 5′-ATCACTGCCACTCAGAAGAC-3′ and 5′-ACATTGGGGGTAGGAACAC-3′. Amplifications were performed, and calibration curves were run in parallel in triplicates for each analysis. Each sample was analyzed six times during each experiment. The experiments were carried out at least twice. Amplification data were analyzed using the Sequence Detector System Software (PE Applied Biosystems). Relative quantification were calculated by normalizing the test crossing thresholds (Ct) with the GAPDH amplified control Ct. The results were normalized to GAPDH and expressed as percentage of controls.

### Soluble RANKL protein secretion assay

Levels of RANKL were determined in culture media supernatants of control cells and cells treated with insulin with commercially available ELISAs (Biomedica, Vienna, Austria).

### Detection of ERK 1/2 and Akt activation in CVSMCs

For studying the effects of insulin on the ERK 1/2 and Akt signaling pathway, Western blot was performed using anti-p-ERK 1/2, ERK 1/2, p-AKT, and AKT as we previous described [39]. The membrane was reprobed with secondary antibody for 1 h. Blots were processed using an ECL kit (Amersham Biosciences) and exposed to the film.

### Measurement of mineralized matrix formation in cultured CVSMCs

Measurment was determined as we previously described [38]. CVSMCs in 24-well plates were cultured in DMEM containing 10% FBS in the presence of 10 nM insulin or vehicle for 12 days. The extent of mineralized matrix was determined by Alizarin Red S staining. Briefly, cells were fixed in 70% ethanol for 1 h at room temperature and stained with 40 mM Alizarin Red S for 10 min. Next, cell preparations were washed with PBS to eliminate nonspecific staining. The stained matrix was photographed using a digital microscope. Alizarin Red S staining was released from cell matrix by incubation in cetyl-pyridinium chloride for 15 min. The amount of released dye was quantified by spectrophotometry at 540 nm. Results were then normalized to total cellular protein values.

### RNA interference for RANKL

RNA interference was used to down-regulate the expression of RANKL in CVSMCs. Two small-interfering RNAs (siRNAs) duplexes targeting rat RANKL gene were synthesized by Genesil Biotechnology (Wuhan, China). The sequences of the sense rat RANKL siRNAs used were 5′-CGCGCUGCUUCUACAGAAUdTdT-3′, these sequences of the antisense rant RANKL siRNA used were 5′-AUUCUGUAGAAGCAGCGCGdTdG-3′ as previously described [40]. The scrambled control siRNA with the same nucleotide composition as the RANKL siRNA but lacking significant sequence homology to the rat genome was also constructed. For gene knockdown experiments, CVSMCs plated in a 60-mm diameter dish and cultured for 24 h in medium without antibiotics. Cells were transfected with siRNAs (1 nmol/well) using Lipofectamine 2000 (Invitrogen Inc., Carlsbad, CA, USA) according to the manufacturer's instructions. After 24 h of culture, cells were retransfected with siRNAs or controls and then recultured for another 48 h. Total RNA was extracted using Trizol reagent (Gibco). Quantitative real-time PCR was performed using Roche Molecular LightCycler (Roche Applied Science, Indianapolis, IN, USA) as described previously.

### Statistical analyses

Data are presented as the mean ± SD. Comparisons were made using a one-way ANOVA. All experiments were repeated at least three times, and representative experiments are shown.
